# A One Health overview, facilitating advances in comparative medicine and translational research

**DOI:** 10.1186/s40169-016-0107-4

**Published:** 2016-08-23

**Authors:** Cheryl Stroud, Igor Dmitriev, Elena Kashentseva, Jeffrey N. Bryan, David T. Curiel, Hans Rindt, Carol Reinero, Carolyn J. Henry, Philip J. Bergman, Nicola J. Mason, Josephine S. Gnanandarajah, Julie B. Engiles, Falon Gray, Danielle Laughlin, Anita Gaurnier-Hausser, Anu Wallecha, Margie Huebner, Yvonne Paterson, Daniel O’Connor, Laura S. Treml, James P. Stannard, James L. Cook, Marc Jacobs, Gerald J. Wyckoff, Lee Likins, Ubadah Sabbagh, Andrew Skaff, Amado S. Guloy, Harlen D. Hays, Amy K. LeBlanc, Joan R. Coates, Martin L. Katz, Leslie A. Lyons, Gayle C. Johnson, Gary S. Johnson, Dennis P. O’Brien, Dongsheng Duan, James P. Calvet, Barbara Gandolfi, David A. Baron, Mark L. Weiss, Debra A. Webster, Francis N. Karanu, Edward J. Robb, Robert J. Harman

**Affiliations:** 1 One Health Commission, Apex, NC 27502 USA; 2 Biologic Therapeutics Center, Washington University in St. Louis, St. Louis, MO 63130 USA; 3 Comparative Oncology, Radiobiology, and Epigenetics Laboratory, University of Missouri, Columbia, MO 65203 USA; 4 Comparative Oncology, Radiobiology, and Epigenetics Laboratory, University of Missouri, Columbia, MO 65211 USA; 5 Comparative Internal Medicine Laboratory, University of Missouri, Columbia, MO 65203 USA; 6 Katonah Bedford Veterinary Center, Bedford Hills, NY 10507 USA; 7 Clinical Studies Division, VCA, Los Angeles, CA 90064 USA; 8 Adjunct Associate Faculty, Memorial Sloan-Kettering Cancer Center, New York City, NY 10065 USA; 9 University of Pennsylvania School of Veterinary Medicine, 3900 Delancey Street, Philadelphia, PA 19104 USA; 10 Office of Professional Studies in the Health Sciences, Drexel University College of Medicine, Room 4801 New College Building, 245 North 15th Street, Philadelphia, PA 19102 USA; 11 Advaxis Immunotherapies Inc., 305 College Road East, Princeton, NJ 08540 USA; 12 ClinData Services Inc., 6713 Holyoke Court, Fort Collins, CO 80525 USA; 13 Department of Microbiology, University of Pennsylvania Perelman School of Medicine, 319A Johnson Pavilion, 3610 Hamilton Walk, Philadelphia, PA 19104 USA; 14 Aratana Therapeutics Inc., Leawood, KS 66211 USA; 15 Department of Orthopaedic Surgery, Missouri Orthopaedic Institute, 1100 Virginia Ave., Columbia, MO 65212 USA; 16 Comparative Orthopaedic Lab, Mizzou BioJoint Center, Missouri Orthopaedic Institute, University of Missouri, Columbia, MO 65211 USA; 17 Musculoskeletal Transplant Foundation (MTF), Edison, NJ 08837 USA; 18 Division of Molecular Biology and Biochemistry, School of Biological Sciences, University of Missouri-Kansas City, Kansas City, MO 64110 USA; 19 Rex Animal Health, Kansas City, KS 66103 USA; 20 Cerner Corporation, Kansas City, MO 64117 USA; 21 Comparative Oncology Program, Center for Cancer Research, National Cancer Institute, National Institutes of Health, Bethesda, MD 20892 USA; 22 Departments of Veterinary Medicine and Surgery, University of Missouri, Columbia, MO 65211 USA; 23 Mason Eye Institute, University of Missouri, Columbia, MO 65211 USA; 24 Veterinary Pathobiology, Comparative Neurology Program, University of Missouri, Columbia, MO 65211 USA; 25 Department of Molecular Microbiology and Immunology, Department of Neurology, Department of Bioengineering, University of Missouri, Columbia, MO 65212 USA; 26 Kidney Institute, University of Kansas Medical Center, Kansas City, KS 66160 USA; 27 Polycystic Kidney Disease Foundation, Kansas City, MO 64114 USA; 28 Department of Anatomy and Physiology, Kansas State University College of Veterinary Medicine, Manhattan, KS 66506 USA; 29 Cardinal Health Regulatory Sciences, Overland Park, KS 64078 USA; 30 Likarda LLC, Kansas City, KS 66103 USA; 31 VetStem Biopharma, Inc., Poway, CA 92064 USA

## Abstract

A1 One health advances and successes in comparative medicine and translational research

Cheryl Stroud

A2 Dendritic cell-targeted gorilla adenoviral vector for cancer vaccination for canine melanoma

Igor Dmitriev, Elena Kashentseva, Jeffrey N. Bryan, David T. Curiel

A3 Viroimmunotherapy for malignant melanoma in the companion dog model

Jeffrey N. Bryan, David Curiel, Igor Dmitriev, Elena Kashentseva, Hans Rindt, Carol Reinero, Carolyn J. Henry

A4 Of mice and men (and dogs!): development of a commercially licensed xenogeneic DNA vaccine for companion animals with malignant melanoma

Philip J. Bergman

A5 Successful immunotherapy with a recombinant HER2-expressing *Listeria monocytogenes* in dogs with spontaneous osteosarcoma paves the way for advances in pediatric osteosarcoma

Nicola J. Mason, Josephine S. Gnanandarajah, Julie B. Engiles, Falon Gray, Danielle Laughlin, Anita Gaurnier-Hausser, Anu Wallecha, Margie Huebner, Yvonne Paterson

A6 Human clinical development of ADXS-HER2

Daniel O’Connor

A7 Leveraging use of data for both human and veterinary benefit

Laura S. Treml

A8 Biologic replacement of the knee: innovations and early clinical results

James P. Stannard

A9 Mizzou BioJoint Center: a translational success story

James L. Cook

A10 University and industry translational partnership: from the lab to commercialization

Marc Jacobs

A11 Beyond docking: an evolutionarily guided OneHealth approach to drug discovery

Gerald J. Wyckoff, Lee Likins, Ubadah Sabbagh, Andrew Skaff

A12 Challenges and opportunities for data applications in animal health: from precision medicine to precision husbandry

Amado S. Guloy

A13 A cloud-based programmable platform for health

Harlen D. Hays

A14 Comparative oncology: One Health in action

Amy K. LeBlanc

A15 Companion animal diseases bridge the translational gap for human neurodegenerative disease

Joan R. Coates, Martin L. Katz, Leslie A. Lyons, Gayle C. Johnson, Gary S. Johnson, Dennis P. O’Brien

A16 Duchenne muscular dystrophy gene therapy

Dongsheng Duan

A17 Polycystic kidney disease: cellular mechanisms to emerging therapies

James P. Calvet

A18 The domestic cat as a large animal model for polycystic kidney disease

Leslie A. Lyons, Barbara Gandolfi

A19 The support of basic and clinical research by the Polycystic Kidney Disease Foundation

David A. Baron

A20 Using naturally occurring large animal models of human disease to enable clinical translation: treatment of arthritis using autologous stromal vascular fraction in dogs

Mark L. Weiss

A21 Regulatory requirements regarding clinical use of human cells, tissues, and tissue-based products

Debra A. Webster

A22 Regenerative medicine approaches to Type 1 diabetes treatment

Francis N. Karanu

A23 The zoobiquity of canine diabetes mellitus, man’s best friend is a friend indeed-islet transplantation

Edward J. Robb

A24 One Medicine: a development model for cellular therapy of diabetes

Robert J. Harman

## A1 One health advances and successes in comparative medicine and translational research

### Cheryl Stroud

#### One Health Commission, Apex, NC, 27502, USA

##### Correspondence: Cheryl Stroud - cstroud@onehealthcommission.org

*Clinical and Translational Medicine* 2016, **5(Suppl 1)**:A1

Antimicrobial resistance, Ebola, *E coli* 0157, MERS, SARS, Salmonellosis, Zika virus and the critical need for yellow fever vaccines have recently emphasized the poignant need for a One Health paradigm shift at all levels of academic, corporate, food production, lawmaking, public policy, and research systems. oAdditionally, the world must soon feed the projected 9 billion people expected to populate its surface without causing further global destruction. Many scientists and One Health advocates believe that a One Health approach and application is the planet’s ‘Ray of Hope’ for the future because they see as unsustainable our current ways of doing business in professional ‘silos’. In addition to human, veterinary and environmental health specialists joining hands, we need anthropologists, chemists, ecologists, educators, engineers, private industry, social scientists, etc. to all work in interactive One Health systems and thinking. This will require conjoined concerted effort.

Over the past decade there has been increasing recognition of just how much can be learned at the interface of human, animal, environmental and plant domains. From heart transplants to management of psychiatric disorders to prosthetic limbs, cancer treatments and vaccine development, tremendous knowledge can be gained when we create opportunities for trans-professional interactions of researchers and clinicians.

This presentation will share a very brief historic context for global efforts to resurrect this very old concept, give an update on current happenings in the One Health ‘movement’ and give examples of how One Health thinking is advancing comparative medicine and translational research.

## A2 Dendritic cell-targeted gorilla adenoviral vector for cancer vaccination for canine melanoma

### Igor Dmitriev^1^, Elena Kashentseva^1^, Jeffrey N. Bryan^2^, David T. Curiel^1^

#### ^1^ Biologic Therapeutics Center, Washington University in St. Louis, St. Louis, MO, 63130, USA; ^2^ Comparative Oncology, Radiobiology, and Epigenetics Laboratory, University of Missouri, Columbia, MO 65203, USA

##### Correspondence: David T. Curiel - DCuriel@radonc.wustl.edu, Jeffrey N. Bryan - bryanjn@missouri.edu

*Clinical and Translational Medicine* 2016, **5(Suppl 1)**:A2

**Background:** To improve the efficacy of adenoviral (Ad) vector vaccines, we have developed an innovative strategy to specifically target Ad vector vaccines to DC. To facilitate targeting of the key DC subsets responsible for CD8 T cell priming, we have developed a strategy to replace the Ad fiber knob with fiber-fibritin chimeras fused to single domain antibodies (sdAb) derived from the camelid family. This innovative strategy ablates the native tropism of Ad and permits selective and efficient DC targeting based on the specificity of the sdAb. Our approach leverages important insights into DC biology that supports targeting of CD8α+/CD141+ DC to enhance active immunization.

**Materials and methods:** Of note, one of the major limits to the use of Ad vector vaccines based on human Ad serotype 5 (huAd5) is the presence of widespread pre-existing immunity in humans. Simian Ad species are closely related but distinct from human Ad. Thus, simian Ad offer unique advantages as vaccine vectors including the ability to efficiently bypass pre-existing immunity to huAd5, while maintaining similar growth properties and genomic structure. To facilitate clinical translation of our innovative Ad vector vaccine platform we propose to engineer and test DC-targeted gorilla Ad (GAd), as we believe that this simian Ad species has the most potential for clinical translation. We have establish a research alliance with GenVec, Inc., which will provide us unique access to novel GAd constructs and reagents to carry out this work.

**Results:** Preliminary data confirms the feasibility and translational potential of our innovative Ad vector vaccine platform. We demonstrate here that (1) we can successfully engineer Ad vector vaccines targeting DC by replacing the fiber knob domain with chimeric fiber-fibritin-sdAb molecules; (2) Ad vector vaccines incorporating DC-specific chimeric fiber-fibritin-sdAb are devoid of native adenovirus tropism, and transduce DC with much greater efficiency; (3) DC-targeted Ad vector vaccines can induce robust immune responses in vivo; (4) we can genetically modify GAd in a maner comparative to huAd5.

**Conclusions:** Our DC targeted Ad vaccines will be evaluated in canine melanoma patients for indication of anti-tumor immunity. Utility in this context will rationalize transition of our targeting modifications to the context of GAd to realize a vaccine with direct clinical utility for canine and human patents.

## A3 Viroimmunotherapy for malignant melanoma in the companion dog model

### Jeffrey N. Bryan^1^, David Curiel^2^, Igor Dmitriev^2^, Elena Kashentseva^2^, Hans Rindt^1,3^, Carol Reinero^3^, Carolyn J. Henry^1^

#### ^1^Comparative Oncology, Radiobiology, and Epigenetics Laboratory, University of Missouri, Columbia, MO, 65211, USA; ^2^Biologic Therapeutics Center, Washington University in St. Louis, St. Louis, MO, 63130, USA; ^3^Comparative Internal Medicine Laboratory, University of Missouri, Columbia, MO 65203, USA

##### Correspondence: David T. Curiel - DCuriel@radonc.wustl.edu, Jeffrey N. Bryan - bryanjn@missouri.edu

*Clinical and Translational Medicine* 2016, **5(Suppl 1)**:A3

**Background:** Melanoma is an aggressive disease with 9940 deaths expected in 2015. The immune system normally protects against development of cancer; however, immune tolerance (a state of antigen unresponsiveness) to melanoma allows progression to a bulky or metastatic state. Outbred companion dogs develop malignant melanoma (CMM) with similar aggressive metastatic traits. The canine immune system both reacts to and is suppressed by similar tumor mechanisms as human, unlike other models. Recent clinical studies have demonstrated incompletely effective anti-tumor immunotherapy efficacy using dendritic cells (DC) vaccines.

**Materials and methods:** Our goal is to construct a modular immunotherapy strategy to stimulate DC to induce potent anti-tumor antigen-specific reactions. We will also develop the canine-specific immunology toolkit to demonstrate this response. The hypothesis is that chimpanzee adenovirus (ChAd) targeted by anti-CD40 camelid single-domain antibody (sdAb) containing a human tyrosinase (huTyr) cassette (a melanoma xeno-antigen) will cause maturation of myeloid DC, elaboration of the xeno-antigen payload, and an anti-tumor immune response.

**Results:** Three client-owned dogs with naturally-occurring CMM will be enrolled in a trial of anti-CD40 sdAb-targeted ChAd. Dogs will have CMM of Stage I–III disease. Following Ad therapy, anti-melanoma immunity will be evaluated using antigen-specific delayed hypersensitivity skin test, immunohistochemistry localization of huTyr on DC in skin biopsies, huTyr-specific IgG by ELISA, and ex vivo cytokine elaboration by huTyr stimulation of lymphocytes.

**Conclusions:** This strategy is expected to generate proof-of-principle for viroimmunotherapy in a highly stringent model of disease. The immunological confirmation of antigen-specific response will facilitate rapid translation to the human clinic.

## A4 Of mice and men (and dogs!): development of a commercially licensed xenogeneic DNA vaccine for companion animals with malignant melanoma

### Philip J. Bergman^1,2,3^

#### ^1^Katonah Bedford Veterinary Center, Bedford Hills, NY, 10507, USA; ^2^Clinical Studies Division, VCA, Los Angeles, CA 90064, USA; ^3^Adjunct Associate Faculty, Memorial Sloan-Kettering Cancer Center, New York, NY 10065, USA

##### Correspondence: Philip Bergman - Philip.Bergman@vca.com

*Clinical and Translational Medicine* 2016, **5(Suppl 1)**:A4

Melanoma is the most common oral malignancy in the dog. Oral and/or mucosal melanoma is generally considered an extremely malignant tumor with a high degree of local invasiveness and metastatic propensity. Median survival times for dogs with oral melanoma treated with surgery are approximately 12–18, 5–9 and 3 months with stage I, II and III disease, respectively. Significant negative prognostic factors include stage, size, evidence of metastasis and a variety of histologic criteria. Standardized treatments such as surgery, coarse-fractionation radiation therapy and chemotherapy have afforded minimal to modest stage-dependent clinical benefits and death is usually due to systemic metastasis. Numerous immunotherapeutic strategies have been employed to date with variable clinical efficacy. Xenogeneic DNA vaccination with a wide variety of targets induces immune responses resulting in generation of Ag-specific T cells and antibodies as well as anti-tumor responses and prolongation of survival. These studies led to a USDA-CVB multi-site clinical trial of xenogeneic human tyrosinase in dogs with stage II or III locally controlled oral malignant melanoma with approval in December, 2009. The use of xenogeneic DNA vaccines appears to represent a leap forward in clinical efficacy. Oral melanoma is a spontaneous syngeneic cancer occurring in outbred, immunocompetent dogs and appears to be the most clinically faithful therapeutic model for human melanoma; further use of canine melanoma as a therapeutic model for human melanoma is strongly encouraged. Our results suggest that veterinary and human cancer centers can effectively collaborate and develop therapeutics which lead to governmental licensure and commercial success.

## A5 Successful immunotherapy with a recombinant HER2-expressing *Listeria monocytogenes* in dogs with spontaneous osteosarcoma paves the way for advances in pediatric osteosarcoma

### Nicola J. Mason^1^, Josephine S. Gnanandarajah^1^, Julie B. Engiles^1^, Falon Gray^1^, Danielle Laughlin^1^, Anita Gaurnier-Hausser^2^, Anu Wallecha^3^, Margie Huebner^4^ and Yvonne Paterson^5^

#### ^1^University of Pennsylvania School of Veterinary Medicine, 3900 Delancey Street, Philadelphia, PA 19104, USA; ^2^Office of Professional Studies in the Health Sciences, Drexel University College of Medicine, Room 4801 New College Building, 245 North 15th Street, Philadelphia, PA 19102, USA; ^3^Advaxis Inc. 305 College Road East, Princeton, NJ 08540, USA; ^4^ClinData Services Inc., 6713 Holyoke Court, Fort Collins, CO 80525, USA; ^5^Department of Microbiology, University of Pennsylvania Perelman School of Medicine, 319A Johnson Pavilion, 3610 Hamilton Walk, Philadelphia, PA 19104, USA

##### Correspondence: Nicola J. Mason - nmason@vet.upenn.edu

*Clinical and Translational Medicine* 2016, **5(Suppl 1)**:A5

**Background:** Osteosarcoma is an aggressive, mesenchymal bone tumor that affects 400 children annually. Standard of care includes amputation and chemotherapy however, 30 % of patients die from metastatic disease. No therapeutic advances have been made in osteosarcoma for 30 years. Pet dogs spontaneously develop osteosarcoma that shares similar clinical, biological and molecular features with pediatric osteosarcoma. Canine standard of care involves amputation and chemotherapy, however, the majority of dogs die from metastatic disease within 1 year. We have taken a comparative approach to determine whether a recombinant HER2 expressing *Listeria* (*Lm*-LLO-chuHER2) can prevent the development of metastatic osteosarcoma.

**Materials and methods:** 18 dogs that had undergone amputation and chemotherapy for HER2 + appendicular osteosarcoma were recruited to a phase 1, 3 + 3 dose escalation clinical trial. After chemotherapy, dogs received *Lm*-LLO-chuHER2 intravenously three times, at 3 week intervals. Dogs were evaluated for adverse events, immune responses against HER2 and progression free survival and overall survival.

**Results:** Repeat doses of up to 3.3 × 10^9^ CFU of *Lm*-LLO-chuHER2 were well tolerated. 15/18 dogs developed immune responses against HER2 within 6 months of immune therapy. Median progression free survival was 615 days and overall survival was 965 days compared to overall survival of a historical control group that was 420 days.

**Conclusions:** Administration of *Lm*-LLO-chuHER2 is safe, induces HER2-specific immune responses and prolongs progression free and overall survival in dogs with osteosarcoma. These promising results pave the way for a clinical trial to determine the effectiveness of *Lm*-LLO-chHER2 in preventing metastatic disease in pediatric osteosarcoma.

## A6 Human clinical development of ADXS-HER2

### Daniel O’Connor

#### Advaxis Immunotherapies, Inc., Princeton, NJ 08540, USA

##### Correspondence: Daniel O'Conner - OConnor@advaxis.com

*Clinical and Translational Medicine* 2016, **5(Suppl 1)**:A6

ADXS-HER2 is an *Lm* Technology™ immunotherapy product candidate being developed by Advaxis to target HER2 expressing cancers. ADXS-HER2 has received orphan drug designation by the U.S. Food and Drug Administration (FDA) and the European Medicines Agency (EMA) for the treatment of osteosarcoma. Advaxis is developing ADXS-HER2 for both human and animal health, and has seen encouraging data in canine osteosarcoma, which is considered a model for human osteosarcoma.

Dr. Nicola Mason, PhD, BVetMed, associate professor of medicine at the University of Pennsylvania School of Veterinary Medicine, evaluated the immunogenicity, safety, and impact of attenuated, recombinant Listeria monocytogenes (Lm) transformed with a HER2/Neu fusion protein (ADXS-HER2) on survival in 18 dogs with surgically treated osteosarcoma. Advaxis has licensed ADXS-HER2 to Aratana Therapeutics, Inc. for the development of pet therapeutics and expects that the HER2 construct will be conditionally approved in 2016 to treat dogs with osteosarcoma.

Human epidermal growth factor receptor 2 (HER2) is overexpressed in a percentage of solid tumors such as gastric, bladder, brain, pancreatic, ovarian and pediatric bone cancer (osteosarcoma). HER2 expression is associated with more aggressive disease, increased risk of relapse and decreased overall survival, and is an important target for immunotherapy.

## A7 Leveraging use of data for both human and veterinary benefit

### Laura S. Treml

#### Aratana Therapeutics, Inc., Leawood, Kansas 66211, USA

##### Correspondence: Laura S. Treml - ltreml@aratana.com

*Clinical and Translational Medicine* 2016, **5(Suppl 1)**:A7

Naturally-occurring diseases in client-owned dogs and cats often mimic diseases that occur in humans. Because pet animals are outbred and share their environment with humans, disease in these animals is thought to be a better model of human disease than those that can be produced in laboratory animals. Therefore, data produced in studies in veterinary patients can often promote human development programs. This data can also be used as part of the data package submitted to regulatory agencies for veterinary products.

Advaxis, Inc. is developing a *Listeria monocytogenes*-based therapeutic vaccine for use in humans with osteosarcoma. Aratana Therapeutics, which licensed the product from Advaxis, is developing the same therapeutic vaccine for use in dogs with osteosarcoma, seeking licensure for this product from the United States Department of Agriculture, Center for Veterinary Biologics (USDA-CVB). Immunotherapies most often fall under the jurisdiction of USDA-CVB and the agency requires specific information about the manufacturing, safety and effectiveness of all products that they govern. Effectiveness data generated to support human development programs can be used to support the regulatory requirements for veterinary use.

## A8 Biologic replacement of the knee: innovations and early clinical results

### James P. Stannard

#### Department of Orthopaedic Surgery, Missouri Orthopaedic Institute, 1100 Virginia Ave., Columbia, MO 65212, USA

##### Correspondence: James P. Stannard - stannardj@health.missouri.edu

*Clinical and Translational Medicine* 2016, **5(Suppl 1)**:A8

Articular cartilage injuries are a major cause of pain and dysfunction in the knee. Translational research in this area led to a number of innovations and the formation of the Mizzou Biojoint Center. This presentation will focus on the innovations and early clinical results from the Mizzou Biojoint Center. Several patients have experienced remarkable clinical improvement allowing them to resume an active lifestyle.

Several breakthroughs have occurred as a result of the partnership between the Missouri Orthopaedic Institute and the Comparative Orthopaedics Laboratory at the University of Missouri. We have determined that at least 70% of the chondrocytes in a transplanted specimen must be viable in order to achieve success. We have also analyzed the ideal size and shape for grafts that are being transplanted and determined that a thickness of only 6–7 mm is ideal to balance strength with diffusion characteristics. We have also determined that soaking the donor graft in the recipients bone marrow aspirate concentrate exponentially speeds the process of boney incorporation of the graft. Finally, we have determined that storage in a special media at room temperature yields grafts with better chondrocyte survival at 60 days than chondrocyte survival with conventional storage methods at 28 days.

Early results of the Biojoint articular cartilage transplants demonstrate improvements in function scores, International Knee Documentation Committee scores, VAS pain scores, and the promis mobility score. The most remarkable improvements occurred at 1 year following the procedure. Several examples of clinical cases will be discussed.

Biologic replacement of full thickness articular cartilage changes has undergone dramatic changes at the Univesity of Missouri as a result of a unique translational partnership between the Missouri Orthopaedic Institute and the Comparative Orthopaedics Laboratory.

## A9 Mizzou BioJoint Center: a translational success story

### James L. Cook

#### Comparative Orthopaedic Lab, Mizzou BioJoint Center, Missouri Orthopaedic Institute, University of Missouri, Columbia, MO 65211, USA

##### Correspondence: James L. Cook - cookjl@health.missouri.edu

*Clinical and Translational Medicine* 2016, **5(Suppl 1)**:A9

The Mizzou BioJoint Center is a sub-specialty clinical service dedicated to providing cutting edge treatments for joint problems. These treatments are designed to restore patients’ function and quality of life. At the center, physicians and scientists work together on a daily basis to find optimal, evidence-based solutions for the most common joint disorders affecting society today.

The Mizzou BioJoint Center was birthed from translational research performed under the One Health–One Medicine umbrella, which is a hallmark focus of research at the University of Missouri. Research in the Comparative Orthopaedic Lab that was initially focused on dogs and horses proved to be very safe and effective in veterinary medicine and so the process of translation to human application was pursued. This process involved a team of veterinarians, physicians, and scientists working together to develop techniques and instrumentation for preserving and implanting allograft tissues for joint restoration through a series of in vitro, ex vivo and animal model studies. This translational research came to fruition in terms of validation of pre-clinical safety and efficacy based on FDA, ASTM and AATB standards, and was subsequently funded for first-in-man trials. Several of the technologies involved in the BioJoint process have been licensed and are now being used commercially as well.

Initial results in human patients have been excellent overall and the Mizzou BioJoint Center has already become an internationally-recognized center of excellence for delivering innovative restorative solutions for the joint problems experienced by millions of people worldwide. Importantly, this concept is still helping veterinary patients as well.

## A10 University and industry translational partnership–from the lab to commercialization

### Marc Jacobs

#### Musculoskeletal Transplant Foundation (MTF), Edison, NJ 08837, USA

##### Correspondence: Marc Jacobs - marc_jacobs@mtf.org

*Clinical and Translational Medicine* 2016, **5(Suppl 1)**:A10

MTF is a registered and certified, non-profit tissue bank that recovers, prepares, and distributes allograft tissue forms worldwide for surgical use in multiple orthopaedic specialities, wound care, and general and plastic reconstructive surgery. MTF provides peer reviewed Foundation Grants and Directed Research Grants to support the advancement of allograft science and product development partnerships.

Successful translation of proprietary university research to commercial applications can take many paths. The MIZZOU/MTF partnership began with a review of MIZZOU proprietary technology for fresh osteochondral allografts, intellectual property, and in vitro and in vivo data provided by MIZZOU. MTF considered the surgeon and patient benefits of the technology, the Regulatory pathway and potential reimbursement requirements, synergy of the MIZZOU and MTF goals, and MTF’s competencies to actively support commercial development and product distribution in its due diligence review. A partnership was created through a Sponsored Research Agreement (SRA) funded by MTF that defined the development activity protocols, resources, and safety and efficacy outcomes required to translate the technology to commercialization. An Option to License the MIZZOU intellectual property including predetermined licensing terms was created simultaneously with the SRA.

SRA activity focused on process development and tissue supply by MTF and in vitro measurements at MIZZOU. Successful completion of the SRA has resulted in initiation of an approved patient assessment study and limited distribution of the allograft tissue to select sites to create journal publications that will describe the health care and patient efficacy benefits of the technology.

## A11 Beyond docking: an evolutionarily guided OneHealth approach to drug discovery

### Gerald J. Wyckoff, Lee Likins, Ubadah Sabbagh, Andrew Skaff

#### Division of Molecular Biology and Biochemistry, School of Biological Sciences, University of Missouri-Kansas City, Kansas City, MO 64110, USA

##### Correspondence: Gerald J. Wyckoff - wyckoffg@umkc.edu

*Clinical and Translational Medicine* 2016, **5(Suppl 1)**:A11

Solving the problems inherent in modern drug discovery will require more than just changing how we are going to examine small molecules and potential targets. It instead will require a completely different mindset that recognizes why drugs fail to come to market, how we can utilize data integrated across human and animal health, and a precision medicine approach designed to discover new targets and lead compounds- possibly before the conditions the target proteins underlie are even understood clinically. The advent of clinical whole genome sequencing means that the average genetic fitness load carried by individuals—the sum of their proclivity towards disease when encountering certain environmental factors- will start to be revealed. We analyzed data from publically available sources and carried out statistical analysis in R. Our work shows that genes which code for the proteins which are the targets of most successful drugs are quite evolutionarily conservative- especially when compared to those genes which are causal for disease. In addition, orphan diseases in Humans have cognates in both companion and farm animals which can be studied and developed to help further drug development while at the same time expanding the market for new drugs. We conclude that a OneHealth approach towards finding new drug targets, and associated small molecules, is warranted- from both a market perspective and an evolutionary one- to maximize successful drug development for future precision medicine implementations. We highlight potential novel methodologies for finding these targets that will require further development.

**Acknowledgements:** The authors would like to thank Ada Solidar (B-tech Consulting). This work was funded in part by subcontracts to Dr. Wyckoff from NIH 2 R44 GM097902-02A1 (Dockhorn, PI) and NIH 1 R21 AI113552-01 (Geisbrecht, PI).

**Competing interests:** Gerald J. Wyckoff has an equity stake in Zorilla Research, LLC.

## A12 Challenges and opportunities for data applications in animal health: from precision medicine to precision husbandry

### Amado S. Guloy

#### Rex Animal Health, Kansas City, KS 66103, USA

##### Correspondence: Amado S. Guloy - amado@rexanimalhealth.com

*Clinical and Translational Medicine* 2016, **5(Suppl 1)**:A12

The use of “big data” in healthcare is becoming more widely accepted especially with the announcement of the precision medicine initiative. The increase in adoption of electronic health records, genomics/proteomics/transcriptomics/etc. databases, and the aggregation of environmental data has opened up doors with which researchers can truly understand disease transmission and pathogenesis.

Animal health databases tend to be far more robust than even the most robust human health databases. The opportunity for the development of precision medicine and precision husbandry is wide open, but there remains many challenges standing in the way. This presentation aims to cover the challenges and opportunities that are left in this wide open space.

## A13 A cloud-based programmable platform for health

### Harlen D. Hays

#### Cerner Corporation, Kansas City, MO 64117, USA 

##### Correspondence: Harlen D. Hays - harlen.hays@cerner.com

*Clinical and Translational Medicine* 2016, **5(Suppl 1)**:A13

*HealtheIntent* is a multi-purpose, programmable platform designed to scale at a population level while facilitating health and care at a person and provider level. This cloud-based platform enables health care systems to aggregate, transform and reconcile data across the continuum of care. *HealtheIntent* collects data from multiple, disparate sources in near real-time, providing clarity to millions of data points in an actionable and programmable workflow. It enables organizations to identify, score and predict the risks of individual patients, allowing them to match the right care programs to the right individuals.

This presentation will focus on the ways in which a platform such as this could be used to accelerate the adoption curve of new research and connect disparate sources of information to provide a more complete picture of health and wellness for populations. Through integration of geospatial information, it will be possible to create bridges between human and animal health that can be used at the point of care. Special attention will be paid to how this will enable further research and integration of new knowledge.

**Competing interests:** Mr. Hays is an employee of Cerner Corporation, the provider of the *HealtheIntent* platform.

## A14 Comparative oncology: One Health in action

### Amy K. LeBlanc

#### Comparative Oncology Program, Center for Cancer Research, National Cancer Institute, National Institutes of Health, Bethesda, MD 20892, USA

##### Correspondence: Amy K. LeBlanc - amy.leblanc@nih.gov

*Clinical and Translational Medicine* 2016, **5(Suppl 1)**:A14

Cancer researchers have a unique opportunity to include naturally occurring cancers in pet dogs as translational models in the development path of new human cancer drugs, to the ultimate benefit to both species. Naturally occurring cancers in pet dogs and humans share many features, including histological appearance, tumor genetics, molecular targets, biological behavior, and response to both conventional and novel targeted cancer therapies. Formal integration of studies including pet dogs with cancer is becoming a more common component of an innovative cancer drug development process. Because no gold standard treatments exist for pet animals with cancer, new treatments can be provided to pet dogs with cancer at earlier stages of progression than human trials. Flexibility in the conduct and design of such trials permits serial collection of biologic samples and imaging endpoints during exposure to novel cancer agents. Lastly, cancer progresses at a faster rate in pet dogs than in humans; accordingly, these studies can be completed without interrupting the existing development path. The intramural NCI’s Center for Cancer Research established the comparative oncology program (COP) to develop this novel cancer drug development opportunity, address potential risks with this approach, and establish the organizational infrastructure to undertake translational clinical trials in pet dogs. This clinical trial infrastructure unites study sponsors with 22 academic veterinary centers to support multicenter clinical trials of investigational therapeutics. In this presentation we provide examples of such trials and the unique aspects of drug development that are addressed within the conduct and design of the trial.

## A15 Companion animal diseases bridge the translational gap for human neurodegenerative disease

### Joan R. Coates^1^, Martin L. Katz^2^, Leslie A. Lyons^1^, Gayle C. Johnson^3^, Gary S. Johnson^3^, Dennis P. O’Brien^1^

#### ^1^Departments of Veterinary Medicine and Surgery, ^2^Mason Eye Institute, ^3^Veterinary Pathobiology, Comparative Neurology Program, University of Missouri, Columbia, MO 65211, USA

##### Correspondence: Joan R. Coates - CoatesJ@missouri.edu

*Clinical and Translational Medicine* 2016, **5(Suppl 1)**:A15

Many inherited neurodegenerative diseases in our companion animals share important similarities to human diseases in terms of clinical signs, pathology and genetics and may prove to be excellent models of these conditions. Veterinary researchers play a critical role in characterization of the natural history of inherited neurologic diseases in animals. Recent advances in molecular genetics allow for rapid identification of the mutation underlying the disease and correlation with the genetic basis of the corresponding human disease. Disease mutation discovery enables further understanding of pathophysiologic mechanisms and ultimately development of rational approaches to therapy. *Thus, a naturally*-*occurring canine disease model offers a ready clinical population where therapies can be evaluated in a setting closely mimicking human clinical trials.*

Therapeutic studies using transgenic rodent models often fail to predict efficacy and outcome in human patients. As an intermediate-sized model of a naturally-occurring neurodegenerative diseases, dogs will yield data more relevant to the human disease. Similarities between the canine and human nervous systems in size and complexity, and the homogeneity in onset and clinical progression of many canine diseases facilitate therapy translation. Moreover, utilizing established disease measures in canine models provide sensitive and specific milestones of disease progression and therapeutic response that parallels surrogate markers used in human patients. Importantly, canine disease models permit studies of therapy intervention using similar procedures as those in human patients. Demonstrating dosing and delivery paradigms and safety of therapy in the canine disease models will provide key supportive data and improve the probability of clinical trial success.

## A16 Duchenne muscular dystrophy gene therapy

### Dongsheng Duan

#### Department of Molecular Microbiology and Immunology, Department of Neurology, Department of Bioengineering, University of Missouri, Columbia, MO 65212, USA

##### Correspondence: Dongsheng Duan - duand@health.missouri.edu

*Clinical and Translational Medicine* 2016, **5(Suppl 1)**:A16

Duchenne muscular dystrophy (DMD) is caused by dystrophin deficiency. Gene therapy holds a great promise to restore dystrophin expression and ameliorate muscle disease. Adeno-associated virus (AAV) is currently the leading vector for DMD gene therapy. However, AAV can only carry a 5-kb genome while the full-length dystrophin coding sequence is 11.2-kb. To overcome this hurdle, investigators have developed highly abbreviated synthetic mini- and micro-dystrophin genes. The minigenes are 6 to 8-kb in length. They evolve from a 6.2-kb naturally occurring minigene that results in ambulation to the age 61. We have developed a series of dual and tri-AAV vectors that can deliver the minigene and even the full-length dystrophin cDNA. In dual vectors, the minigene expression cassette is split to two parts and individually packaged in an AAV vector. Reconstitution is achieved in muscle through engineered recombination signals. Dual AAV minigene delivery has resulted in saturated transduction following direct muscle injection and widespread transduction after intravenous injection in mouse models of DMD. A microgene carries only one-third of dystrophin cDNA. We have recently engineered novel microgenes that can anchor neuronal nitric oxide synthase (nNOS) to the sarcolemma. We further demonstrated therapeutic efficacy in mouse models of DMD. In preparation for human translation, we recently tested AAV microgene therapy in the canine model. Direct muscle injection ameliorated histopathology and improved muscle function. Importantly, a single intravenous injection resulted in whole body micro-dystrophin expression and amelioration of histological lesions. Our results have set the foundation for bodywide gene therapy in DMD patients.

**Acknowledgements:** Our studies are supported by NIH, DOD, Jesse’s Journey, MDA, PPMD and Hope for Javier.

**Disclosure:** Dongsheng Duan is a member of the scientific advisory board for and an equity holder of Solid GT, a subsidiary of Solid Biosciences.

## A17 Polycystic kidney disease: cellular mechanisms to emerging therapies

### James P. Calvet

#### Kidney Institute, University of Kansas Medical Center, Kansas City, KS 66160, USA

##### Correspondence: James P. Calvet - jcalvet@kumc.edu

*Clinical and Translational Medicine* 2016, **5(Suppl 1)**:A17

Autosomal dominant polycystic kidney disease (ADPKD) is a proliferative disorder in which thousands of cysts develop from renal tubules, enlarging over decades of time to eventually overwhelm the kidneys. Cyst formation in PKD involves both neoplastic cell growth and cyst-filling fluid secretion. Secondary effects of cyst growth including inflammatory infiltrates and fibrosis contribute to renal failure. Murine genetic models of PKD have been instrumental for mutation analysis, structure–function studies, and for elucidating the mechanisms of cyst formation, growth and enlargement, and disease progression, and have served as models for the development of therapies to slow cyst growth and kidney enlargement.

The protein products of the ADPKD genes PKD1 and PKD2 are polycystin-1 and polycystin-2, which are thought to form a membrane-associated receptor-channel complex that regulates intracellular calcium. Decreasing intracellular calcium can bring about a phenotypic switch causing renal epithelial cells to abnormally proliferate in response to cyclic AMP agonists. By manipulating cellular calcium or cAMP levels it is possible to stimulate cell proliferation and fluid secretion, and in mouse and rat models to affect disease severity. Insights derived from such studies have led to the development of therapies to decrease cAMP and ameliorate cystic disease. One therapy, the vasopressin receptor antagonist, tolvaptan, was successfully tested in a phase 3 clinical trial and is awaiting federal approval. Other therapies based on this idea are in the preclinical or clinical trials pipeline.

## A18 The domestic cat as a large animal model for polycystic kidney disease

### Leslie A. Lyons, Barbara Gandolfi

#### Departments of Veterinary Medicine and Surgery, University of Missouri, Columbia, MO 65211, USA

##### Correspondence: Leslie A. Lyons - lyonsla@missouri.edu

*Clinical and Translational Medicine* 2016, **5(Suppl 1)**:A18

Autosomal dominant polycystic kidney disease (ADPKD) is a commonly inherited disorder that causes the formation of fluid-filled renal cysts leading to renal failure. ADPKD is more common than sickle cell anemia and muscular dystrophy combined. Mutations in the gene *polycystin*–1 (*PKD1)* cause 85 % of ADPKD. The *PKD1* gene is very large and most families with ADPKD have new mutations in the gene, thus, many different mutations cause disease. Feline PKD is also autosomal dominant and has clinical presentations similar to humans. While some cats have severe and rapid disease, dying by 4–6 years, many cats have less prominent disease and live long lives. PKD affects ~38 % of Persian cats worldwide, which is approximately 6 % of cats, making it the most prominent inherited feline disease. The feline version of *PKD1* has a mutation that causes a loss of ~25 % of the tail end of the protein. The same mutation has not been identified in humans, although similar regions of the protein are truncated. Like humans, only one copy of the mutation is needed to cause this severe disease and no individuals have two copies of the mutation because it causes death of the developing embryo. Only recently, potential effective treatments for PKD been discovered. Because the cat’s disease is so similar to humans, cats can be used to test the efficacy and longer-term effects of these new drugs. Therefore, *domestic cats can be a valuable naturally*-*occurring model to evaluate treatments and therapies for humans with polycystic kidney disease.*

## A19 The support of basic and clinical research by the Polycystic Kidney Disease Foundation

### David A. Baron

#### Polycystic Kidney Disease Foundation, Kansas City, MO 64114, USA

##### Correspondence: David A. Baron - davidb@pkdcure.org

*Clinical and Translational Medicine* 2016, **5(Suppl 1)**:A19

The Polycystic Kidney Disease (PKD) Foundation was founded in 1982 to promote research into the mechanisms of this most common, potentially fatal genetic renal disease, and facilitate the development of novel therapeutics to mitigate the progression of PKD and its systemic pathophysiological consequences. After the NIH, the PKD Foundation is the second largest source of research funds for the two forms of PKD, autosomal dominant (ADPKD) and autosomal recessive (ARPKD). ADPKD, which affects approximately 1/500 live births is caused by mutations in either the *PKD1* or *PKD2* genes that code for polycystin-1 and polycystin-2, respectively. ARPKD, which is diagnosed in about 1/20,000 live births is caused by mutations in *PKHD1*, which codes for fibrocystin. These proteins are associated with the cilium, an organelle present in renal tubular cells, as well as many other cell types in the body. PKD, therefore, is sometimes referred to as a ciliopathy.

Renal cysts grow inexorably for years or decades, causing the destruction of adjacent normal kidney parenchyma and in many cases ultimately cause end stage renal disease, which then necessitates renal replacement therapy—dialysis or kidney transplant. The PKD Foundation is devoted to finding new therapeutic approaches to slow or prevent cyst formation and growth. No specific therapy for PKD is currently approved in the U.S.

The PKD Foundation currently awards 15 two-year basic and clinical research grants and five two-year basic and clinical research fellowships in nephrology every other year to develop novel therapies and the next generation of PKD scientists and clinicians.

## A20 Using naturally occurring large animal models of human disease to enable clinical translation: treatment of arthritis using autologous stromal vascular fraction in dogs

### Mark L. Weiss

#### Kansas State University College of Veterinary Medicine, Dept of Anatomy and Physiology, Manhattan, KS 66506, USA

##### Correspondence: Mark L. Weiss - weiss@vet.k-state.edu

*Clinical and Translational Medicine* 2016, **5(Suppl 1)**:A20

Osteoarthritis (OA) is the most common form of arthritis and affected about 26.9 million US adults in 2005 at a cost of approximately $60B. Based on 2003 NHIS projections, about 67 million (25 %) adults aged 18 years or older will have doctor-diagnosed arthritis by the year 2030 at a cost that may exceed $220B. OA is also the most common source of chronic pain in dogs, with 20–50 % of dogs over the age of three affected. For this reason, naturally occurring OA in dogs should be evaluated for its potential to model human OA. Owing to the number of aging baby boomers and OA’s socioeconomic impact, it is urgent to improve OA animal models for testing novel therapeutics for translational medicine. Here, we evaluate autologous adipose-derived stromal vascular fraction, called SVFs. SVF contains beneficial cells, cytokines and growth factors and is easily obtained from adults. SVF offers a plethora of distinct advantages e.g., rapid isolation, autologous sourcing, and simple preparation with no need to expand in vitro, large number of cells, wide availability, and is conducive to fast clinical deployment.

To test SVF, we conducted an FDA-registered INAD. Design: randomize, placebo controlled, double blind clinical study using 22 dogs with OA seen at the Kansas State University College of Veterinary Medicine. SVF was collected from all patients, and processed using a commercial protocol which has been used in veterinary medicine for >2 years. Outcome assessment was done using clinical assessment by blinded clinical observer or quality of life and function assessment by the blinded owners using previously validated clinical instruments. Quantitative outcome assessment was done using pressure sensitive walkway. SVF was found to be safe for treating canines with OA. SVF was found to improve function with moderate to large standardized effect size. This pilot study provided effect size estimates, effectiveness and safety endpoints that support a fully powered phase III trial. This works supports the contention that naturally occurring OA in a large animal model can enable translational medicine.

## A21 Regulatory requirements regarding clinical use of human cells, tissues, and tissue-based products

### Debra A. Webster

#### Cardinal Health Regulatory Sciences, Overland Park, KS 64078, USA

##### Correspondence: Debra A. Webster - debra.webster@cardinalhealth.com

*Clinical and Translational Medicine* 2016, **5(Suppl 1)**:A21

Human cells, tissues, and tissue-based products (HCT/Ps) have long been regulated by FDA under section 361 of the Public Health Services (PHS) Act and 21 CFR Part 1271, using a risk-based approach aimed at preventing the introduction, transmission, and spread of communicable diseases. Human tissues are solely regulated under the PHS Act provided they meet certain criteria. Two criteria, minimal manipulation and homologous use, have been the subject of increased discussion and enforcement as the use of HCT/Ps has expanded beyond implantation, transplantation, infusion, or transfer into a human recipient. Minimal manipulation is codified as processing that does not alter the original relevant characteristics of the tissue. Homologous use is codified as repair, reconstruction, replacement, or supplementation of a recipient’s cells or tissues with an HCT/P that performs the same basic function(s) in the recipient as in the donor. The definitions of minimal manipulation and homologous use and their interpretations have come under challenge with the recent advances in the use of HCT/Ps in regenerative medicine, and have far-reaching consequences for current manufacturers of and companies developing HCT/Ps for clinical use. If an HCT/P does not meet the criteria set forth in 21 CFR 1271.10(a), and the establishment that manufactures the HCT/P does not qualify for any of the exceptions listed in 21 CFR 1271.15, then the HCT/P will be regulated under section 351 of the PHS Act and will require premarket clearance or a valid marketing license as a drug, biologic, or medical device.

## A22 Regenerative medicine approaches to type 1 diabetes treatment

### Francis N. Karanu

#### Likarda LLC, Kansas City, KS 66103, USA

##### Correspondence: Francis N. Karanu - fkaranu@likarda.com

*Clinical and Translational Medicine* 2016, **5(Suppl 1)**:A22

Diabetes is a chronic metabolic disorder affecting humans as well as several species of domestic animals and pets. In humans, type 1 diabetes is a multifactorial autoimmune disease involving progressive destruction of pancreatic beta cells ultimately resulting in absolute lack of insulin. Type 1 diabetes is treated by injections of exogenous insulin, a complicated process requiring elaborate blood glucose monitoring and dosage selection. This has spurred efforts to seek alternative forms of treatment. Two key approaches now in use or in clinical trials include cadaveric islet transplants and use of stem cell derived insulin producing beta-like cells capable of correcting the diabetic condition. Islet cell replacement therapy is complicated and requires intensive patient immunosuppression, often leading to other complications. Moreover, the paucity of enough cadaveric islets limits this as a long term approach to diabetes treatment. Thus, alternative sources of inexhaustible insulin producing cells are needed. Several groups have reported success with creating insulin producing cells from pluripotent stem cells (embryonic or induced pluripotent stem cells), capable of treating diabetes in animal models. These reports have facilitated commencement of the first FDA approved clinical trials in humans. Among the key challenges for this approach are product safety, potency, protection of transplants from the recipient immune response requiring encapsulation of transplanted cells, as well as determination of appropriate transplant sites that promote effective vascularization. Successful resolution of these issues with long term independence from insulin injections will pave the way for successful treatments for millions of diabetes patients and their family pets.

## A23 The zoobiquity of canine diabetes mellitus, man’s best friend is a friend indeed-islet transplantation

### Edward J. Robb

#### Likarda LLC, Kansas City, KS 66103, USA

##### Correspondence: Edward J. Robb - erobb@likarda.com

*Clinical and Translational Medicine* 2016, **5(Suppl 1)**:A23

Canine diabetes is classified as insulin dependent diabetes mellitus similar to type II diabetes in man both characterized by hypoinsulinemia, with failure of glycemic control. Peak prevalence in dogs is at 7–10 years, females are twice as frequent. Keeshonds, schnauzers and terrier breeds are at higher risk. Overall prevalence of 0.5–1.0 %. Presentation includes polydipsia, polyuria, polyphagia and weight loss. Well tolerated cases can present with cataracts. Persistent fasting hypoglycemia and glycosuria are diagnostic. Commercial insulins, veterinary or human ‘label ‘are typically administered twice a day. Dose adjustments are based on urine glucose levels and blood glucose via point of care glucometers. Diet and exercise are emphasized. Oral hypoglycemic drugs are not utilized as cases present with hypoinsulinemia.

Recurrence or persistence of clinical signs is common, suggestive of insulin ineffectiveness but is most likely associated with owner compliance/technique. Control in dogs vs. man is characterized as underdoing due to concerns of hypoglycemia. Many owners upon diagnosis elect euthanasia because of compliance, or later in the course due to the burden of care, hypoglycemia and cataract s/blindness.

There are over 600 scientific publications where dogs were the preclinical model for human islet transplantation, and in greater than 250 publications, dogs were recipients. Utilizing this scientific base to provide a therapeutic solution back to dogs is zoobiquitious. Today, novel tissue-processing technologies allow for cell-based therapies (CBT) without immunosuppression. CBT offers improved glycemic control, convenience, and curtails progression of complications (blindness) while strengthening the bond on both ends of the leash.

**Competing interests:** Edward J. Robb is a Consultant to Likarda.

## A24 One medicine: a development model for cellular therapy of diabetes

### Robert J. Harman

#### VetStem Biopharma, Inc., Poway, CA, 92064, USA

##### Correspondence: Robert J. Harman - bharman@vetstem.com

*Clinical and Translational Medicine* 2016, **5(Suppl 1)**:A24

The emergence of cell-based regenerative therapy has broadened the concept of “one medicine” from zoonotic diseases to one of shared biology. Diabetes is a disease common to human and companion animals. In dogs, the high prevalence, onset, diagnosis and management are quite similar to that in the human. Cell-based therapeutic approaches include (1) islet transplantation, (2) creation of new islet cells to transplant, and (3) mesenchymal stem cell therapy to down-regulate autoimmunity and stimulate regeneration of beta cells.

The development pathway for human and veterinary products consists of preclinical models, safety and efficacy testing, and manufacturing development. The veterinary FDA regulators (Center for Veterinary Medicine) and human regulators (Center for Biologics Evaluation and Research) follow generally common pathways to approval of cell therapy products. Historically, the dog has been used as a preclinical model, but with emergence of clinical veterinary regenerative medicine, in-clinic experiences with naturally-occurring diabetes will benefit the development of new therapies. The regulatory challenges include donor sourcing and disease screening, manufacturing methods development and validation, and clinical testing requirements. Each of the three approaches above will require custom designed development and regulatory programs.

All mammals share similar stem cell biology and the clinical, experimental, manufacturing learnings should be shared to expedite market approval in all species. Veterinary clinical data can be used as more realistic predictors of cell therapy outcomes in human clinical studies and reduce the cost and time to develop new therapies for dogs and humans.

**Competing interests:** Robert J. Harman is an employee of VetStem Biopharma.

